# Revisiting chemical kinetics for H + CH_4_ → H_2_ + CH_3_ using variational transition state theory: comparison with global dynamics calculations

**DOI:** 10.1007/s00894-026-06930-1

**Published:** 2026-09-09

**Authors:** Edson F. V. de Carvalho, Marcelo A. P. Pontes, Luiz F. A. Ferrão, Francisco B. C. Machado, Orlando Roberto-Neto

**Affiliations:** 1 Coordenação Do Curso de Licenciatura Em Física, Universidade Federal Do Maranhão, São Luís, Maranhão Brazil; 2 Departamento de Engenharia, Faculdade Anhanguera de Jacareí, Jacareí, São Paulo Brazil; 3 Departamento de Química, Instituto Tecnológico da Aeronáutica, São José Dos Campos, São Paulo Brazil; 4 Laboratório de Computação Científica Avançada e Modelamento (Lab – CCAM), Instituto Tecnológico da Aeronáutica, São José Dos Campos, São Paulo Brazil; 5 Divisão de Aerotermodinâmica E Hipersônica, Instituto de Estudos Avançados, São José Dos Campos, São Paulo Brazil

**Keywords:** Direct and reverse abstraction reactions, ωB97X-D surface, M06-2X surface, CCSD(T), Kinetic isotope effect

## Abstract

**Context:**

For both the forward and reverse reaction, there are no accurate rate constants at the VTST/MT level using potential energy surfaces built with the density functional theory (DFT) approach. Calculations of rate constants in function of temperature are carried out for the elementary reaction H + CH_4_ → H_2_ + CH_3_ using variational transition state theory with multidimensional tunneling corrections (VTST/MT) with density functional theory (DFT) and are compared with previous global dynamics calculations reported in the literature. Calculations show that the dual-level method based on an M06-2X PES is capable of providing an accurate framework to compute thermal rate constants in comparison with global dynamics methods.

**Methods:**

The CCSD(T) method with the aug-cc-pVnZ (*n* = 3–5) basis set is employed to characterize the stationary states for the forward and reverse reactions and the M06-2X and ωB97X-D DFTs are used to generate potential energy surfaces (PESs) in the VTST/MT calculations. At the CVT/μOMT level and using the M06-2X PES, the rate constants at 300 and 600 K are 2.54 × 10^−19^ and 2.92 × 10^−15^ cm^3^ molecule^−1^ s^−1^, which are in excellent agreement with the multi-configurational time-dependent Hartree (MCTDH) calculations (1.9 × 10^−19^ and 2.9 × 10^−15^ cm^3^ molecule^−1^ s^−1^). For the reverse H_2_ + CH_3_ reaction, CVT/μOMT shows good agreement with results using CCSD(T) and analytical PESs. The kinetic isotope effect (KIE) (k^CH4+H^/k^CH4+D^) at 500, 600, and 700 K are 0.65, 0.71, and 0.77 in good agreement with experimental data, respectively equal to 0.8 ± 0.2, 0.9 ± 0.2, and 1.0 ± 0.2.

**Supplementary Information:**

The online version contains supplementary material available at https://doi.org/10.1007/s00894-026-06930-1.

## Introduction

Accurate chemical dynamics studies are computationally demanding since they scale exponentially with the degrees of freedom, and the magnitude of total angular momentum [1]. Conversely, statistical mechanics methods such as the variational transition state theory (VTST) are formally simpler approach and computationally cheaper than global dynamics, as quantum dynamics (QD) and quasi-classical trajectory theory (QCT) [2]. This occurs because VTST does not require full knowledge of the potential energy surface (PES) or of the excited electronic states [3]. The VTST-MT model assumes that kinetics properties are mainly dependent on the regions of the potential energy surface close to the stationary points (reactant, saddle point, products) and to the intrinsic reaction coordinate [1, 3, 4], while also relying on the validity of the fundamental assumptions of the transition state theory (TST), namely local equilibrium and the validity of Maxwell–Boltzmann distribution [5].

Usually, TST calculations overestimate the rate constants which are associated with the vibrational degrees of freedom (DOFs) and higher density of states at the saddle point, which make the statistical distribution of energy difficult among the DOFs within a relatively short collision time. This limitation was simpler interpreted as the large re-crossing at the transition state [2]. There are few revisions comparing the VTST approach with global dynamics which include quantum dynamics (QD), quasi-classical trajectory (QCT) [2, 6], and ring polymer molecular dynamics (RPMD) [7]. As far as we know, all VTST calculations are based on global analytical potential energy surfaces [1–10].

For the forward abstraction reaction CH_4_ + H → H_2_ + CH_3_, Espinosa-Garcia [9] and Pu and Truhlar [10] have compared VTST calculations with exact quantum dynamics (QD) results on the analytical PESs. The error at the CVT/μOMT level is approximately 21% in relation to the QM method at 200 K and it was considered very encouraging, particularly because temperatures in the range of 200–300 K, for instance, are highly relevant for atmospheric chemistry modeling [1].

The CH_4_ + H → H_2_ + CH_3_ is one of the main elementary reactions of the global CH_4_/O_2_ combustion mechanism [11] and is a prototype of polyatomic system in theoretical [1–10, 12–21] and experimental [22–24] (https://kinetics.nist.gov/kinetics/Detail?id=2001SUT/SU669-684:1; https://kinetics.nist.gov/kinetics/Detail?id=2021BUR/MAN023102:1; https://kinetics.nist.gov/kinetics/Detail?id=1992BAU/COB411-429:109; https://kinetics.nist.gov/kinetics/Detail?id=2001SUT/SU669-684:2; https://kinetics.nist.gov/kinetics/Detail?id=2019PEA/AYE1861-1873:6 (NIST 2019) https://kinetics.nist.gov/kinetics/Detail?id=1991RAB/SUT674-681:3) (NIST 1991) studies. Theoretical studies have been employed ab initio and analytical global potential energy surfaces in variational transition state theory with multidimensional tunneling (VTST/MT) calculations [3, 4, 9, 10], quasiclassical trajectory (QCT) [2, 6, 9], ring polymer molecular dynamics (RPMD) [7], and multiconfigurational time-dependent Hartree (MCTDH) calculations [16–19]. Xu et al. [18] provided a review of the global potential energy surface (PES) including the Jordan–Gilbert JG (PES) [25, 26], an ab initio PES based on Shepard interpolation approach, and the neural network (NN) [18] fitted to ab initio calculations. In contrast, fewer studies have focused on the H_2_ + CH_3_ → CH_4_ + H reaction [4, 12, 14].

The VTST/MT methods have been based on modified Jordan–Gilbert PJ (PES), and Shepard interpolation approach analytical surfaces [8, 9, 14, 17]. Those global PESs have classical barrier height varying from 10.92 to 15.4 kcal mol^−1^ and imaginary harmonic frequencies at the transition state ranging from 1093 to 1500 cm^−1^ with the electronic reaction energy varying from 1.7 to 3.6 kcal mol^−1^ [14], which correspond to a thermoneutral process of ΔH_0_ = 0.03 ± 0.01 kcal mol^−1^ [1] (Table 1). As far as we know, for both the forward and reverse reaction, there are no accurate rate constants at the VTST/MT level using potential energy surfaces built with the density functional theory (DFT) approach.

**Table 1:** Forward barrier height (${V}^{\ddagger }$) (kcal mol^−1^), reaction energy (ΔE) (kcal mol^−1^), and the normal-mode imaginary frequency at the saddle point (cm^−1^), and enthalpy of the bond energy of abstraction reaction (ΔH_0_) of H + CH_4_ → CH_3_ + H_2_ (R1)

Methods	${V}^{\ddagger }$	ΔE	ΔH_0_	ω_i_
ωB97X-D/aug-cc-pVTZ	13.56	4.42	1.36	1310i
M06-2X/aug-cc-pVTZ	15.56	3.66	2.60	1539i
M08-HX/aug-cc-pVTZ	15.37	3.89	0.93	989i
BB1K/aug-cc-pVTZ	14.59	4.78	1.46	1248i
CCSD(T)/aug-cc-pVTZ	14.71	2.89	− 0.38	1452i
CCSD(T)/aug-cc-pVQZ	14.77	2.80	− 0.32	1469i
CBS_T-Q_	14.81	2.73	− 0.39	
^b^CCSD(T)/aug-cc-pV5Z	14.82	2.81	− 0.31	
^a^Exp.:			0.03 ± 0.1	

^a^[1, 48] Computed with Hess’s law; ^b^CCSD(T)/aug-cc-pV5Z//CCSD(T)/aug-cc-pVQZ

In the present study, the thermal rate constants of the forward (CH_4_ + H) and reverse rate (H_2_ + CH_3_) elementary reactions were provided employing the VTST/MT approach for which the potential energy surfaces are built by the M06-2X [27] and ωB97X-D [28] DFT methods. These DFT approximations have been successfully employed in our previous studies involving hydrogen abstraction reactions H + H_2_O_2_ [29] and (H) CH_3_ + SiH_4_ [30]. The present results are compared with previous VTST, global dynamics calculations, and with experimental fitting data [22, 23]. Calculations of kinetic isotope effects, *k*^H^/*k*^D^ (KIEs), are also carried out since it provides a sensitive test for the barrier heigh, zero-point energy (ZPE), and tunneling effects. The following reaction paths are investigated,

R1 $$H+CH_4\rightarrow H_2+CH_3$$

R2 $$H_2+CH_3\rightarrow H+\:CH_4$$

R3 $$H+CD_4\rightarrow HD+CD_3$$

R4 $$D+CH_4\rightarrow HD+CH_3$$

## Methodology

The geometry and harmonic frequencies of the stationary points (reactant, products, and first-order saddle point) were characterized through the minimization of the ground-state electronic energy using the M06-2X [27, 31] and ωB97X-D [28] functionals, as well as the CCSD(T) method [32] employing the aug-cc-pVTZ and aug-cc-pVQZ basis sets [33]. Single-point calculations at the CCSD(T)/aug-cc-pV5Z were also carried out using CCSD(T)/aug-cc-pVQZ geometries. Also, the CCSD(T) with aug-cc-pVTZ and aug-cc-pVQZ values of barrier high and reaction energy were estimated to the complete basis set (CBS) limit using the scheme of Halkier et al. [34].

The minimum energy paths (MEP) in isoinertial coordinates were scaled to the reduced mass μ and the signed distance along this path is labeled as *s* [15]. By convention, *s* = 0 indicates the location of the transition state, whereas *s* > 0 and s < 0 correspond to reactants and products’ sides, respectively [35]. The MEPs were computed from − 1.2 Å (reactants side) to 1.0 Å (products side) for (R1) and (R2) reaction paths. The multireference character of the reactants, transition states, and products for both (R1) and (R2) reactions was assessed by using the *T*_1_ diagnostic [36]. *T*_1_ values larger than 0.02 for closed-shell and larger than 0.045 for open-shell systems indicated a multireference character of the wave function [37]. The electronic structure calculations were carried out with the Gaussian 09 [38] quantum chemistry software package.

Thermal rate constants were calculated with variational transition state theory with interpolated optimized corrections (VTST-IOC) [38]. This dual-level method involves interpolating a low-level reaction energy path with high-level electronic energies and vibrational frequencies [35]. The low-level paths were calculated using the M06-2X/aug-cc-pVTZ and ωB97X-D/aug-cc-pVTZ methods.

The ground-state thermal rate constants (*k*^CVT/G^(T)) were computed using the VTST method [3] which locates the dividing surface along the reaction coordinate (*s*) at the maximum of the free energy of activation ($\Delta {G}^{GT,0}(T,{s}_{*}^{CVT})$),

5 $$k^{CVT\;/G}\left(T\right)=\sigma\frac{k_BT}hK^0\;exp\left[-\triangle G^{GT,\;0}\left(T,s_\ast^{CVT}\right)/k_BT\right]\;$$

in which σ is the reaction-path symmetry number of reaction [39], ${k}_{B}$ is the Boltzmann constant, and ${K}^{0}$ is the reciprocal of the standard-state concentration in units of cm^3^molecule^−1^ s^−1^. The rate constant calculations were carried out at the temperature range of 200 to 2000 K. 

To introduce quantum effects on the motion along the reaction coordinate, ${k}^{CVT/G}\left(T\right)$ is multiplied by a ground-state transmission coefficient, $K^{CVT,G}$, accounting for the tunneling and nonclassical reflection effects. The quantized rate constant is given by

6 $${k}^{CVT/G}\left(T\right)={\kappa }^{CVT/G}{k}^{CVT/G}$$

The methods used to calculate the transmission’s coefficients (${\kappa }^{CVT/G}$) include multidimensional zero-curvature tunneling (ZCT) [40, 41], small-curvature tunneling (SCT) [40, 42], large-curvature tunneling (LCG4) [15], and microcanonically optimized multidimensional tunneling (μOMT) [43]. In the μOMT method, at each total energy, the larger of the SCT and LCT tunneling probabilities is accepted as the best estimate. Thermal rate constants were carried out with the POLYRATE code [44] and the GAUSSRATE interface [45] for potential energy surfaces calculations.

A procedure to estimate the reaction-path curvature is to compute the skew angle (*β*) between the entrance and exit valley of potential energy surface [46] and Garret and Truhlar [47] have compiled collinear three-atoms reactions between the values of skew angle and the transmission coefficients of hydrogen abstraction reactions of the type A + BC → AB + C, where A, B, C, and C may be atoms or group of atoms.

In the study of various MEPs, this rule predicts that larger values of the skew angle, i.e., close to 90 degrees indicate the predominance of small-curvature tunneling and smaller values closer to ~ 0 degree indicate large-curvature tunneling effects due to the presence of swath paths on the potential energy surface. The skew angle is defined by [46]

7 $$\beta =arccos{\left(\frac{{m}_{A}{m}_{C}}{{m}_{AB}{m}_{BC}}\right)}^{1/2}$$

where A, B, and C may be atoms or group of atoms. The values of skew angles for (R1), (R2), (R3), and R4 are respectively, 46.7, 56.9, and 37.3 37.7° suggesting an intermediate reaction-path curvature to (R1) and (R2) with predominance of small multidimensional tunneling and more important large-curvature effects for the deuterated H + CD_4_ → HD + CD_3_ (R3) and D + CH_4_ → HD + CH_3_ (R4) reactions. For comparison, the benchmark hydrogen abstraction reaction Cl + CH_4_ → CH_3_ + HCl [48], which chemical kinetics is strongly controlled by large-curvature multidimensional tunneling, has a skew angle equal to 16.3 degrees.

## Results

### Molecular structures and energetics

The *T*_1_ diagnostic [36] of the reactants, transition state, and products computed at CCSD(T)/aug-cc-pVQZ geometries presented values below 0.02 (Table 1S). The values for CH_5_ (saddle point), CH_4_, CH_3_, and H_2_ are 0.016, 0.009, and 0.005, respectively. These results are below the limit recommended by Rienstra-Kiracofe et al. [37] and indicate that all species are predominantly monoconfigurational.

The Cartesian coordinates of the optimized geometries and zero-point corrected electronic energies (ZPVE) of reactants, products, and transition structures with CCSD(T), M062X, and ωB97X-D methods and the aug-cc-pVTZ and aug-cc-pVQZ basis sets are provided in Table 2S. Tables 3S to 7S listed the values of harmonic vibrational frequencies of hydrogen and deuterated species H_2_, CH_4_, CH_3_, and CH_5_ (first-order saddle point). Table 1 lists energetics data including the forward barrier height (${V}^{\ddagger }$) (kcal mol^−1^), reaction energy (ΔE) (kcal.mol^−1^), the enthalpy of the bond energy at 0 K (ΔH_0_), and the normal-mode imaginary frequency at the saddle point (ω_i_). The value of ω_i_ gives the curvature of the PES in the reaction coordinates at the saddle point and affects the curvature shape on the entrance channel of the reaction; therefore, it is crucial parameter for both VTST/MT [1, 3] and accurate molecular dynamics [2].

Our best calculations of barrier height and reaction energy are 14.8 and 2.8 kcal mol^−1^ computed at the CCSD(T)/aug-cc-pV5Z//CCSD(T)/aug-cc-pVQZ level, which are the same range of previous high-level ab initio calculations, i.e., 14.8 to 15.1 [4, 9]. Also, this method gives value of ΔH_0_ of − 0.31 kcal mol^−1^ in closer agreement experimental value (0.03 ± 0.1 kcal mol^−1^) computed with Hess’s law [48]. Calculations with the BB1K [31] and M08-HX [49] methods were not considered to build the PES because their harmonic frequencies, related to the width the PES close to the transition state, differ by 159.06 and 482.14 cm^−1^ in relation to the CCSD(T)/aug-cc-pVQZ method.

### Rate constants

**Fig. 1:**
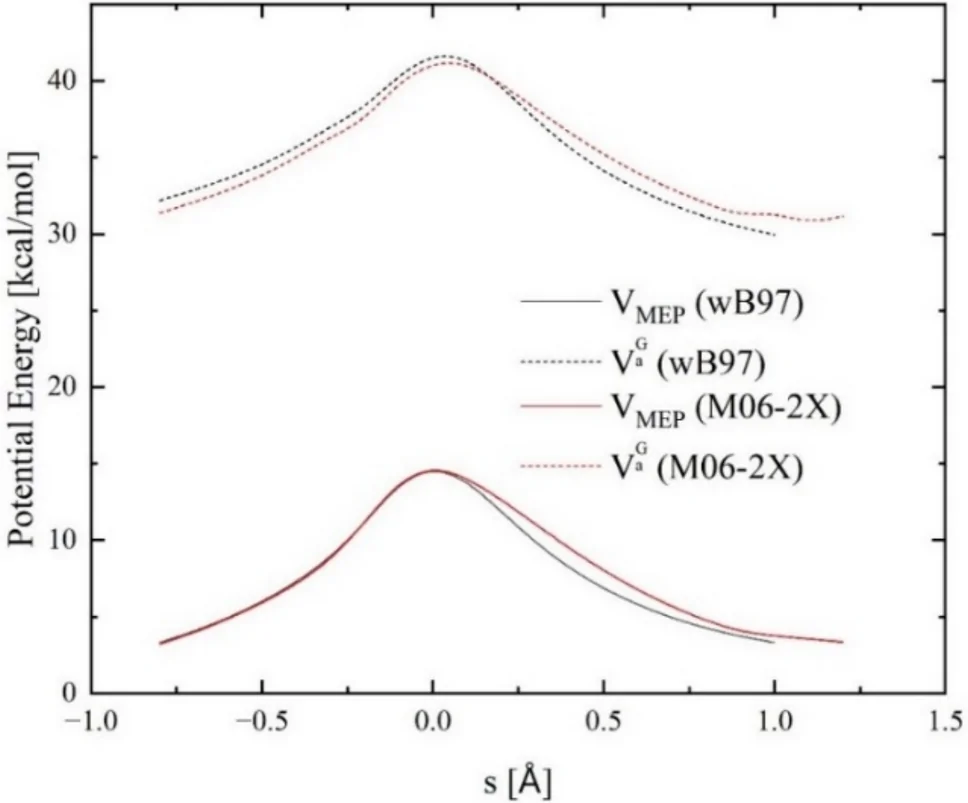
Electronic and vibrational adiabatic potentials of CH_4_ + H → H_2_ + CH_3_ using the ωB97X-D and M06-2X functionals

Table 2 gives thermal rate constants computed by the VTST-IOC method at the CVT/μOMT level, using PESs built by the ωB97X-D and M06-2X D functionals. The CCSD(T)/aug-cc-pVTZ harmonic frequencies are employed as high-level (HL) corrections [35]. Tables 8S to 13S include values of rate constants computed by CVT, CVT/SCT, and CVT/μOMT methods. Tables 14S to 19S list rate constants computed using the ωB97X-D and M06-2X harmonic frequencies named low-level (LL) corrections. Figure 1 shows the electronic and vibrational adiabatic potentials of CH_4_ + H → H_2_ + CH_3_ using the ωB97X-D and M06-2X functionals.

**Table 2:** Rate constants (in cm^3^ molecule^−1^ s^−1^) of CH_4_ + H → H_2_ + CH_3_ (*k*_1_) reaction computed with CVT/μOMT previous calculations and experimental data

T (K)	Present study		Previous studies employing analytical PESs					Theory	Experimental	
	ωB97X-D	M06-2X	ICVT/μOMT^9^	CVT/μOMT^10^	CVT/LAT^15^	RPMD^7^	MCTDH^19^	*d*-TST^21^	Fit-2001^22^	Fit-2021^23^
200	3.22E-21	1.99E-22			1.87E-22			1.40E-23		
250	1.00E-19	1.17E-20		4.0E-21				4.89E-21		
300	1.33E-18	2.54E-19	5.03E-19	1.1E-19	5.06E-19	4.73E-19	1.9E-19	3.08E-19	1.90E-19	3.10E-19
350	1.03E-17	2.85E-18					2.4E-18	5.75E-18	2.26E-18	
400	5.45E-17	1.98E-17	4.16E-17	1.2E-17	4.18E-17	3.93E-17	1.8E-17	7.23E-17	1.82E-17	2.86E-17
500	7.10E-16	3.64E-16	6.96E-16	2.6E-16	6.96E-16	6.51E-16	3.6E-16	2.27E-15	3.33E-16	4.87E-16
600	4.69E-15	2.92E-15	5.00E-15	2.2E-15	5.00E-15	4.60E-15	3.0E-15	2.44E-14	2.57E-15	3.50E-15
700	2.00E-14	1.40E-14				2.03E-14		1.39E-13		1.52E-14
800	6.34E-14	4.82E-14	6.72E-14	4.1E-14	6.28E-14	6.28E-14		5.23E-13	3.99E-14	4.75E-14
900	1.63E-13	1.31E-13				1.58E-13		1.49E-12		1.19E-13
1000	3.59E-13	3.00E-13	3.58E-13	2.6E-13	3.58E-13	3.31E-13		3.34E-12	2.43E-13	2.57E-13
1500	4.85E-12	4.44E-12	4.20E-12	4.1E-12	4.20E-12	3.72E-12		1.37E-11	3.80E-12	3.15E-12
1550	5.83E-12	5.36E-12		4.9E-12						
1800	1.29E-11	1.21E-11							1.10E-11	8.15E-12
1950	1.91E-11	1.81E-11							1.71E-11	1.20E-11
2000	2.16E-11	2.05E-11	1.70E-11	1.9E-11	1.70E-11	1.49E-11

Table 2 also includes previous results calculated with ICVT/μOMT [9], CVT/μOMT [10], and CVT/LCT4 [15], ring polymer molecular dynamics (RPMD) [7], and multi-layer multi-configurational time-dependent Hartree (MCTDH) [17]. All these methods employ various levels of global analytical PES. Rate constants computed by the *deformed*-transition state theory [21] method and experimental data [22, 23] are also listed. Calculations of skew angle of (R1) (46.7°), (R2) (56.9°), (R3) (37.3°), and R4 (37.7°) indicate relatively small predominance of swath tunneling thus expected small to intermediate values of multidimensional tunneling.

In the present CVT/μOMT calculations, the values of barrier height and reaction energy are set to 14.82 and 2.81 kcal mol^−1^ respectively. One drawback to the previous VTST, MCTDH, and RPMD studies is that they employ global PES based on different values of energetic parameters and imaginary frequency. Values of barrier height vary from 10.9 to 15.1 kcal mol^−1^ [1, 3, 13]; the reaction energy from 1.7 to 2.9 kcal mol^−1^, and the harmonic transition state frequency vary from 1293 to 1500 cm^−1^.

For the forward CH_4_ + H reaction (R1), the CVT/μOMT rate constants computed with the M06-2X PES are in good agreement with recent fitted equation of rate constants [23] across a wide temperature range (300–1950 K) (Table 2). The recommended fitted rate expression of Burguess et al. [23] is statistically equivalent to earlier fitted data by Sutherland et al. [22] but agrees slightly better with high-temperature and low-temperature data.

Calculations are also in excellent agreement with multi-configurational time-dependent Hartree (MCTDH) [19] results. For instance, at 300 and 600 K, CVT/μOMT values are 2.54 × 10^−19^ and 2.92 × 10^−15^ cm^3^ molecule^−1^ s^−1^ in agreement with the MCTDH calculations 1.9 × 10^−19^ and 3.0 × 10^−15^ cm^3^ molecule^−1^ s^−1^, respectively. Ju and Zhang [2] commented that an explanation for a good agreement between VTST and MCTDH of H + CH_4_ reaction is that both theoretical models employ the normal-mode approximation to represent the Hamiltonian and that the 21% of the vibrational energy is coupled to reaction coordinate and 79% is uncoupled; therefore, most of the reactive vibration modes act as just spectator modes.

**Fig. 2:**
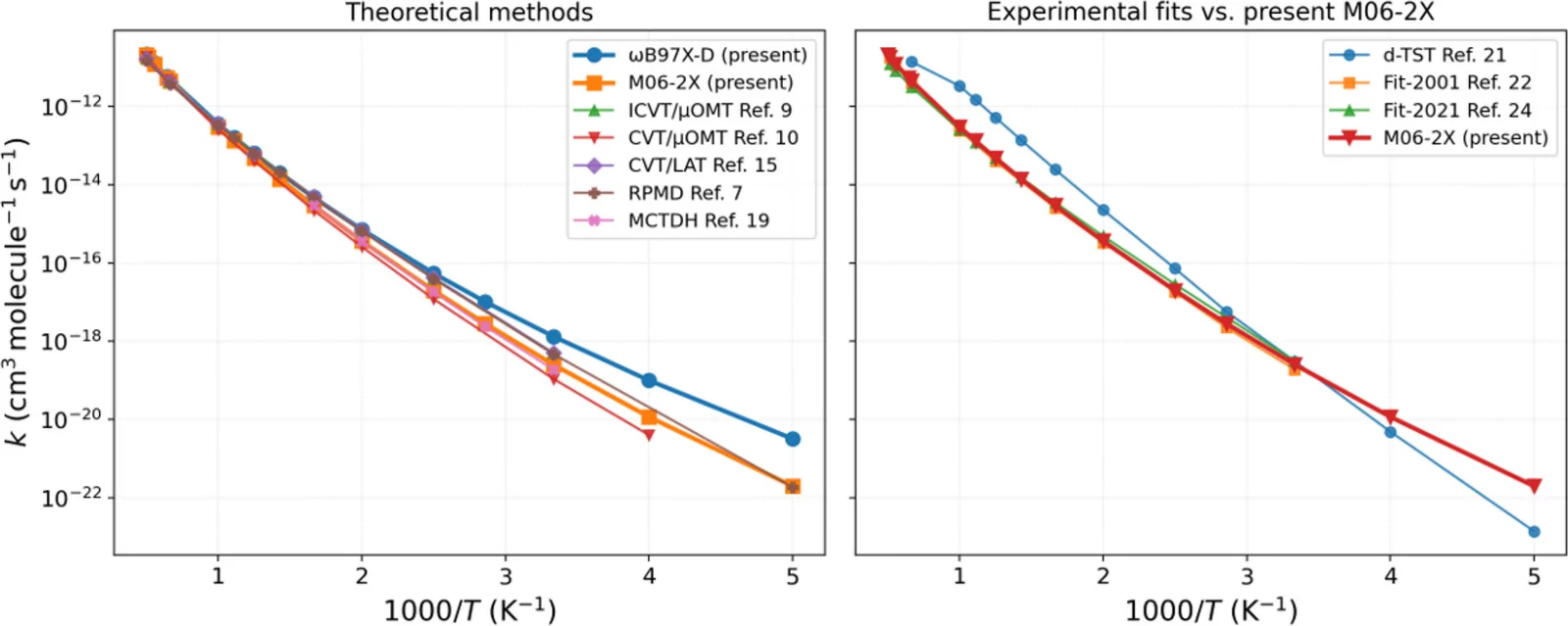
Arrhenius plots of theoretical and experimental rate constants of CH_4_ + H → H_2_ + CH_3_ reaction. Rate constants are given in cm^3^ molecule^−1^ s^−1^ as a function of 1000/T (K^−1^)

Figure 2 shows Arrhenius plot of CVT/μOMT computed by M06-2X and ωB97X-D approaches and experimental data for (R1). Table 8S shows that CVT/μOMT rate constants, corrected by M062-2X frequencies, are in excellent agreement with accurate calculations carried out by the RPMD method in the temperature range of 300 to 1000 K and agree with the more recent recommended data [23]. Otherwise, VTST/μOMT rate constants corrected by CCSD(T)/aug-cc-pVTZ frequencies (Table 8S) are in better agreement with MCTDH calculations and with fitted experimental data. Thermal rate constants calculated using the ωB97X-D surface (Table 2) are slightly higher at low temperatures.

CVT/μOMT rate constants obtained by Espinosa-Garcia [9] using global PESs for (R1) are in excellent agreement with results obtained by M06-2X surface corrected by the M06-2X aug-cc-pVTZ harmonic frequencies (Table 14S) in all range of temperatures. For instance, at 300, 500, and 1000 K, the rate constants are 5.0 × 10^−19^, 6.96 × 10^−16^, and 3.58 10^−13^ cm^3^ molecule^−1^ s^−1^ [2] and 4.87 × 10^−19^, 5.14 × 10^−16^, and 3.60 × 10^−13^ cm^3^ molecule^−1^ s^−1^ (present study). There is large difference in the CVT/μOMT rate constants obtained by Espinosa-Garcia [9] and Pu and Truhlar [10] using slightly different global PES (Table 8S), i.e., at 300, 600, and 1000 K and the ratio between then are 8.6, 4.6, and 2.7. This discrepancy computed with slightly close PESs could be related to the form of symmetrical system of the analytical global PES (Scheme 1 in reference [9]). Thermal rate constants computed by the *d*-TST [21] seems reliable only at low temperatures.

In Fig. 2, the left graph presents the CVT/μOMT rate constants computed with the M06-2X and ωB97X-D potential energy surfaces, compared with previous VTST calculations based on global analytical PESs (ICVT/μOMT [9], CVT/μOMT [10], CVT/LAT [15]), and global-dynamics methods (RPMD [7], MCTDH [19]). The right graph presents M06-2X rate constants compared with the deformed transition-state theory results (d-TST [21]) and the recommended experimental rate expressions (Fit-2001 [22] Fit-2021 [23]).

**Fig. 3:**
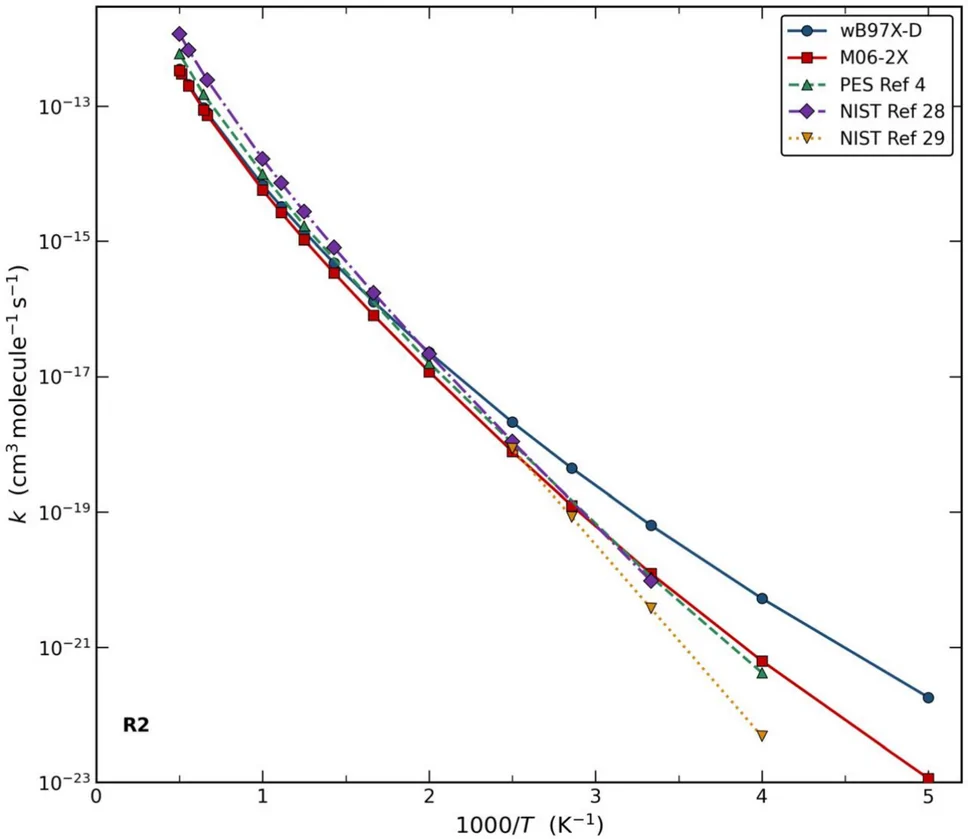
Arrhenius plot of theoretical and experimental rate constants of H_2_ + CH_3_ → CH_4_ + H reaction. Rate constants are given in cm^3^ molecule^−1^ s^−1^ as a function of 1000/T (K^−1^)

Theoretical and experimental data of H_2_ + CH_3_ → H_2_ + CH_4_ reaction (R2) is given in Table 3. CVT/μOMT rate constants calculated with the M06-2X DFT are in better agreement than those ones computed with the ωB97X-D DFT and the rate constants computed by an analytical global PES built by the CCSD(T)/cc-pVTZ methodology [4]. Experimental data (https://kinetics.nist.gov/kinetics/Detail?id=1991RAB/SUT674-681:3) (NIST 1991) seems inconsistent with the theoretical calculations. The Arrhenius plot containing theoretical and experimental rate constants of H_2_ + CH_3_ → CH_4_ + H reaction is given in Fig. 3.

**Table 3:** Rate constants (in cm^3^ molecule^−1^ s^−1^) of reverse CH_3_ + H_2_ → H + CH_3_ reaction (*k*_*-*1_) computed with CVT/μOMT, previous calculations, and experimental data

T(K)	ωB97X-D	M06-2X	PES^1^	NIST 2019NIST^28^	NIST 1991NIST^2^
200	1.81E-22	1.14E-23			
250	5.27E-21	6.25E-22	4.2E-22		4.79E-23
300	6.36E-20	1.23E-20	1.1E-20	9.60E-21	3.76E-21
350	4.43E-19	1.25E-19			8.42E-20
400	2.12E-18	7.82E-19		1.10E-18	8.62E-19
500	2.29E-17	1.19E-17	1.6E-17	2.16E-17	
600	1.29E-16	8.15E-17		1.73E-16	
700	4.83E-16	3.44E-16		8.16E-16	
800	1.38E-15	1.06E-15	1.7E-15	2.74E-15	
900	3.28E-15	2.66E-15		7.31E-15	
1000	6.77E-15	5.73E-15	1.0E-14	1.65E-14	
1500	7.94E-14	7.38E-14		2.44E-13	
1550	9.50E-14	8.87E-14	1.5E-13		
1800	2.09E-13	1.99E-13		6.80E-13	
1950	3.12E-13	3.00E-13			
2000	3.54E-13	3.40E-13	6.0E-13	1.18E-12	

^1^Using an analytical potential energy surface (PES)

Table 4 gives the rate constants and the values of the kinetic isotope effects (*k*^CH4+H^/*k*^CD4+H^) computed by both functionals and they agree with previous calculations using analytical and CCSD(T)/cc-pVTZ PESs [14]. Values of KIEs computed with the M06-2X method and corrected by M06-2X/aug-cc-pVTZ harmonic frequencies (Table 10S) give slightly higher values and computed KIEs by the ωB97X-D approach give slightly lower values.

**Table 4:** Rate constants (in cm^3^ molecule^−1^ s^−1^) of the forward H + CD_4_ → HD + CD_3_ (*k*_2_) and kinetic isotope effects (KIEs) (*k*^CH4+H^/*k*^CD4+H^) computed with CVT/μOMT. KIEs of previous calculations are also shown

T (K)	M06-2X	KIE	ωB97X-D	KIE	PES^14^	CCSD(T)/cc-pVTZ^14^
250	2.46E-22	47.57	1.35E-21	74.07		
300	1.13E-20	22.48	3.67E-20	36.24	25.4	32.1
350	2.25E-19	12.67	5.07E-19	20.31		
400	2.33E-18	8.50	4.25E-18	15.82		
500	7.28E-17	5.00	1.05E-16	6.76		
600	8.13E-16	3.59	1.04E-15	4.51	3.7	3.9
800	1.95E-14	2.47	2.25E-14	2.82		
1000	1.48E-13	2.02	1.63E-13	2.20	2.1	2.1
1500	2.75E-12	1.61	2.88E-12	1.68	1.6	1.6
1800	7.94E-12	1.52	8.22E-12	1.57		
2000	1.38E-11	1.49	1.42E-11	1.52	1.5	1.5

Ref. [14] using global PES

Ref. [14] using CCSDT)/cc-pVTZ

The kinetic isotope effect (*k*^CH4+H^/*k*^CH4+D^) (Table 5) computed with both ωB97X-D and M06-2X approaches is in accordance with previous calculations and experimental data. Considering the ωB97X-D data, the values at 500, 600, and 700 K are 0.65, 0.71, and 0.77 which agree well with experimental data, respectively equal to 0.8 ± 0.2, 0.9 ± 0.2, and 1.0 ± 0.2 [24]. Also, calculations with CVT/SCT [4] and CVT/μOMT [9] methods using different analytical potential energy surfaces give approximately the same results of KIE. Calculations corrected by M06-2X using the aug-cc-pVTZ frequencies (Table 17S) are in excellent agreement with experimental data, i.e., 0.77, 0.82, and 0.86.

**Table 5:** Rate constants (in cm^3^molecule^−1^ s^−1^) of D + CH_4_ → HD + CH_3_ (*k*_3_) and kinetic isotope effect (*k*^CH4+H^/*k*^D+CH4^) computed with CVT/μOMT previous calculations and experimental data

T(K)	M06-2X	KIE	ωB97X-D^1^	KIE	KIE [4]	KIE [9]	Exp. [24]
250	5.50E-20	0.21	2.74E-19	0.36			
300	8.56E-19	0.30	3.02E-18	0.44			
350	7.42E-18	0.38	2.06E-17	0.50			
400	4.28E-17	0.46	9.88E-17	0.55			
500	6.19E-16	0.59	1.10E-15	0.65	0.6	0.71	0.8 ± 0.2
600	4.31E-15	0.68	6.57E-15	0.71	0.7	0.77	0.9 ± 0.2
700	1.89E-14	0.74	2.60E-14	0.77	0.8	0.82	1.0 ± 0.2
800	6.11E-14	0.78	7.84E-14	0.81			
1000	3.52E-13	0.85	4.15E-13	0.87			
1500	4.83E-12	0.92	5.22E-12	0.93			
1800	1.29E-11	0.94	1.36E-11	0.95			
2000	2.17E-11	0.95	2.26E-11	0.96			

Calculations of KIEs (*k*^H2+CH3^/*k*^HD+CH3^) of reverse HD + CD_3_ → H + CD_4_ (Table 6) show that ωB97X-D PES values are slightly better agreement with experimental data, i.e., 2.7, 2.6, and 2.5 versus 2.9, 2.4, and 2.2 [50]. Present CVT/μOMT are also in excellent agreement with VTST and MCTDH calculations, using respectively the PES-2008 [4] (modified Jordan and Gilbert analytical surface) and the Shephard interpolated surface [26].

**Table 6:** CVT/μOMT rate constants (in cm^3^ molecule^−1^ s^−1^) of reverse CH_3_ + HD → D + CH_4_ reaction (*k*_−3_) and kinetic isotope effect (*k*^H2+CH3^/*k*^HD+CH3^), previous calculations, and experimental data

T(K)	M06-2X	KIE	ωb97X-D	KIE	PES-2008 [4]	QM [4, 8]	Exp. [50]
250	3.10E-22	2.01	1.52E-21	3.47			
300	5.83E-21	2.11	2.03E-20	3.13			
350	5.59E-20	2.24	1.53E-19	2.90			
400	3.38E-19	2.31	7.70E-19	2.76	2.7	2.3	2.9
500	5.00E-18	2.36	8.81E-18	2.60	2.6	2.2	2.4
600	3.42E-17	2.38	5.14E-17	2.50	2.5	2.1	2.2
700	1.46E-16	2.36	1.98E-16	2.44	2.4	2.0	
800	4.58E-16	2.31	5.80E-16	2.38			
1000	2.54E-15	2.26	2.95E-15	2.29			
1500	3.45E-14	2.14	3.68E-14	2.16			
1800	9.50E-14	2.09	9.88E-14	2.12			
2000	1.64E-13	2.07	1.69E-13	2.09			

## Conclusion

The equilibrium geometries, harmonic frequencies, and electronic energies of reactants, products, and transition state for the forward H + CH_4_ (R1) and reverse H_2_ + CH_3_ (R2) hydrogen abstraction reactions were computed using the M06-2X, ωB97X-D, and CCSD(T) methodologies. The rate constants and kinetic isotope effects (KIEs) were calculated using variational transition state theory with multidimensional tunneling corrections (VTST/MT) at the VTST-IOC level, using the potential energy surfaces (PESs) derived from ωB97X-D and M06-2X functionals with the aug-cc-pVnZ (*n* = 3–5) basis set. Best values of barrier height (14.82 kcal/mol) and reaction energy (2.81 kcal/mol) were computed with the CCSD(T)/aug-cc-pV5Z method using CCSD(T)/aug-cc-pVQZ geometries. For (R1), the CVT/μOMT computed by the M06-2X PES give excellent agreement with more recent fitted experimental rate constants across a wide temperature range (300–1950 K) [23].

Comparisons with multi-configurational time-dependent Hartree (MCTDH) calculations support the accuracy of the employed approach. For instance, at 300 and 600 K, present CVT/μOMT rate constants using the M06-2X PES values are 2.54 × 10^−19^ and 2.92 × 10^−15^ cm^3^ molecule^−1^ s^−1^, which are in excellent agreement with MCTDH calculations [19], 1.9 × 10^−19^ and 3.0 × 10^−15^ cm^3^ molecule^−1^ s^−1^. For the reverse H_2_ + CH_3_ reaction, CVT/μOMT agree well with previous calculations using the same VTST methodology employing PESs built by both CCSD(T)/cc-pVTZ or using an analytical PES [4]. Rate constants and KIEs computed by the M06-2X PES are in better agreement with quantum mechanical and experimental data than those computed with the ωB97X-D method.

For the forward reaction, the best values of kinetic isotope effects (k^CH4+H^/k^CH4+D^) at 500, 600 and 700 K are 0.77, 0.82, and 0.86 (Table 17S), agree well with experimental data, respectively equal to 0.8 ± 0.2, 0.9 ± 0.2, and 1.0 ± 0.2 [24]. The KIES for the reverse reaction k^CH3+H2^/k^CH3+HD^, at 400, 500, and 600 K, i.e., are 2.31, 2.36, and 2.38, are in agreement with QM (2.3, 2.2, and 2.1) [4, 8] calculations and experimental data (2.9, 2.4, and 2.2) [50].

Through the study of the archetypal CH₄ + H → CH₃ + H₂ reaction, we have shown that, despite the significant advances in potential energy surfaces and reaction dynamics methodologies reported in the literature, dual-level VTST calculations based on the M06-2X potential energy surface achieve comparable accuracy while remaining computationally much more affordable. The calculated thermal rate constants are in excellent agreement with both high-level theoretical results and the available experimental measurements. These findings demonstrate that the dual-level approach provides an accurate and consistent framework for computing thermal rate constants for adiabatic ground-state hydrogen abstraction reactions involving polyatomic species and can be regarded as a reliable benchmark methodology for this class of systems. An additional strength of the present study is the direct validation against experimental rate constants, showing that the dual-level VTST approach not only reproduces high-level theoretical predictions but also accurately describes the measured kinetics.

## Supplementary Information

Below is the link to the electronic supplementary material.

ESM 1 (DOCX 101 KB)

## Data Availability

No datasets were generated or analysed during the current study.
