# Supplementary material for: Revisiting chemical kinetics for H + CH4 → H2 + CH3 using variational transition state theory: comparison with global dynamics calculations
Source: J Mol Model. 2026 Sep 9;32(10):358. doi: 10.1007/s00894-026-06930-1 (PMC13558360; doi:10.1007/s00894-026-06930-1)
Supplement: Supplementary file 1 [file 894_2026_6930_MOESM1_ESM.docx]

**Revisiting Chemical Kinetics for H + CH_4_ → H_2_ + CH_3_ Using Variational Transition State Theory: Comparison with Global Dynamics Calculations**

Edson F. V. de Carvalho^1^, Marcelo A. P. Pontes^2,3,4^ Luiz F. A. Ferrão^3,4^, Francisco B. C. Machado^3,4^ Orlando Roberto-Neto^4,5*^

*^1^Coordenação do Curso de Licenciatura em Física, Universidade Federal do Maranhão, São Luís, Maranhão, Brazil, ^2^Departamento de Engenharia, Faculdade Anhanguera de Jacareí, Jacareí, São Paulo, Brazil, ^3^Departamento de Química, Instituto Tecnológico da Aeronáutica, São José dos Campos, São Paulo, Brazil, ^4^Laboratório de Computação Científica Avançada e Modelamento (Lab – CCAM), Instituto Tecnológico da Aeronáutica, São José dos Campos, São Paulo, Brazil, ^5^Divisão de Aerotermodinâmica e Hipersônica, Instituto de Estudos Avançados, São José dos Campos, São Paulo, Brazil*

**Table 1S** Diagnostic of multi-reference character measured by the *T*_1_ method calculated with the CCSD/aug-cc-pVQZ method.

| Diag | Basis set |  | H | CH_4_ | CH_5_ | CH_3_ | H_2_ |  |
| --- | --- | --- | --- | --- | --- | --- | --- | --- |
|  |  |  |  |  |  |  |  |  |
| *T*_1_ | aug-cc-pVTZ |  | 0.000 | 0.007 | 0.016 | 0.009 | 0.005 |  |
|  |  |  |  |  |  |  |  |  |

**Table 2S** Cartesian coordinates of the optimized geometries, electronic geometries, and zero-point corrected electronic energies (ZPVE) of reactants, products, and transition state computed by the M06-2X, ωB97x-D, and CCSD(T) methods with the aug-cc-pVTZ and set.

**M062X/aug-cc-pVTZ**

**H**

Z	X	Y	Z	(Å)

1	0.000000	0.000000	0.000000

Ee = −0.4982065 a.u.

**H_2_**

Z	X	Y	Z	(Å)

1	0.000000	0.000000	0.369301

1	0.000000	0.000000	-0.369301

E_e_ = -1.1687516 a.u.

E_0_ = -1.158578 a.u.

**CH_3_**

6	0	0.000000	0.000000	0.000000

1	0	0.000000	1.077985	0.000000

1	0	0.933563	-0.538993	0.000000

1	0	-0.933563	-0.538993	0.000000

E_e_ = −39.8253668 a.u.

E_0_ = −39.795502- a.u.

**CH_4_**

6	0.000000	0.000000	0.000000

1	0.000000	0.887638	0.627552

1	-0.887638	0.000000	-0.627000

1	0.000000	-0.887638	0.627552

1	0.887638	0.000000	-0.627552

E_e_ = - 40.5017431 a.u.

E_0_ = -40.456721 a.u.

**CH_5_**

6	0.004597	0.008079	0.001112

1	-0.003250	-0.006589	1.083843

1	0.989489	0.001095	-0.449103

1	-0.700306	0.694764	-0.450889

1	-0.508810	-1.241769	-0.335404

1	-0.849720	-2.042265	-0.542755

Ee = -40.9751583 a.u.

E_0_ = -40.933073 a.u.

**ωB97X-D/aug-cc-pVTZ**

**H**

Z	X	Y	Z	(Å)

1	0.000000	0.000000	0.000000

Ee = -0.5028031 a.u.

**H_2_**

1	0.000000	0.000000	0.371544

1	0.000000	0.000000	-0.371544

E_e_ = −1.1766497 a.u.

E_0_ = −1.166550 a.u.

**CH_3_**

6	0.000000	0.000000	0.000000

1	0.000000	1.077985	0.000000

1	0.933563	-0.538993	0.000000

1	-0.933563	-0.538993	0.000000

E_e_ = −39.8391594 a.u.

E_0_ = −39.809361 a.u.

**CH_4_**

6	0.000000	0.000000	0.000000

1	0.000000	0.888260	0.628097

1	-0.888260	0.000000	-0.628097

1	0.000000	-0.888260	0.628097

1	0.888260	0.000000	-0.628097

E_e_ = −40.5200368 a.u.

E_0_ = −40.475284 a.u.

**CH_5_**

6	-0.027558	-0.048349	-0.045176

1	-0.116005	-0.207288	1.022895

1	0.982187	-0.111442	-0.432992

1	-0.610668	0.779630	-0.430535

1	-0.647249	-1.160758	-0.588105

1	-1.047029	-1.887447	-0.943517

E_e_ = −41.0012585 a.u.

E_0_ = −40.958672 a.u.

**CCSD(T)/aug-cc-pVTZ**

**H**

Z	X	Y	Z	(Å)

1	0.000000	0.000000	0.000000

E_e_ = − 0.4998212 a.u.

**H_2_**

1	0.000000	0.000000	0.371493

1	0.000000	0.000000	-0.371493

E_e_ = −1.1726356 a.u.

E_0_ = −1.162608 a.u.

**CH_3_**

6	0.000000	0.000000	0.000000

1	0.000000	0.000000	1.079494

1	0.934851	0.000000	-0.539737

1	-0.934865	-0.000119	-0.539745

E_e_ = -39.7636609 a.u.

E_0_ = -39.733954 a.u.

**CH_4_**

6	0.000001	0.000005	-0.000006

1	0.123501	0.982506	0.455379

1	-0.084179	-0.754800	0.781756

1	0.863781	-0.221039	-0.626901

1	-0.903108	-0.006699	-0.610196

E_e_ = −40.4409302 a.u.

E_0_ = −40.396130 a.u.

**CH_5_**

6	-0.268968	0.000017	0.000438

1	-0.505762	-0.228282	1.034017

1	-0.523413	1.006360	-0.316767

1	-0.519490	-0.781247	-0.710194

1	1.133058	0.000849	-0.009571

1	2.029415	0.002218	-0.000109

E_e_ = −40.9173029 a.u.

E_0_ = −40.874944 a.u.

**CCSD(T)/aug-cc-pVQZ**

**H**

Z	X	Y	Z	(Å)

1	0.000000	0.000000	0.000000

E_e_ = −0.4999483 a.u.

**H_2_**

1	0.000000	0.000000	0.000000

1	0.000000	0.000000	0.741997

Ee = −1.1738674 a.u.

E_0_ = −1.163842 a.u.

**CH_3_**

6	0.000002	0.000000	-0.000008

1	0.462981	0.973563	0.000017

1	0.611644	-0.887732	0.000017

4	-1.074639	-0.085829	0.000017

E_e_ = −39.773341 a.u.

E_0_ = −39.743550 a.u.

**CH_4_**

6	-0.000003	0.000000	0.000001

1	-0.446785	0.991259	0.044464

1	-0.753720	-0.724324	-0.302461

1	0.387273	-0.267679	0.981084

1	0.813246	0.000746	-0.723094

E_e_ = −40.928132 a.u.

E_0_ = −40.885858 a.u.

**CH_5_**

6	-0.268968	0.000017	0.000438

1	-0.505762	-0.228282	1.034017

1	-0.523413	1.006360	-0.316767

1	-0.519490	-0.781247	-0.710194

1	1.133058	0.000849	-0.009571

1	2.029415	0.002218	-0.000109

E_e_ = −40.9281414 a.u.

E_0_ = −40.885844 a.u.

**Table 3S** Harmonic vibrational frequencies (in cm^−1^) and ZPE (in kcal.mol^−1^) for H_2_, CH_3_, and CH_4_ calculated with the aug-cc-pVTZ^a^ and aug-cc-pVQZ^b^ basis sets.

| System | M062X | ωB97X-D | CCSD(T)^a^ | CCSD(T)^a^ |
| --- | --- | --- | --- | --- |
| H_2_ | 4466.42 | 4434.12 | 4401.74 | 4400.63 |
| ZPE | 6.39 | 6.3375 | 6.292 | 6.291 |
| CH_3_ | 3307.10 | 3309.40 | 3295.43 | 3302.78 |
|  | 3304.38 | 3307.84 | 3295.11 | 3302.55 |
|  | 3128.20 | 3127.48 | 3115.25 | 3119.46 |
|  | 1409.75 | 1411.19 | 1419.10 | 1421.03 |
|  | 1408.16 | 1402.83 | 1418.76 | 1420.36 |
|  | 418.87 | 506.26 | 496.25 | 510.50 |
| ZPE | 18.55 | 18.67 | 18.64 | 18.69 |
| CH_4_ | 3165.80 | 3168.40 | 3146.81 | 3154.48 |
|  | 3163.34 | 3168.30 | 3146.54 | 3154.31 |
|  | 3162.90 | 3165.56 | 3146.33 | 3154.01 |
|  | 3049.08 | 3049.51 | 3027.53 | 3032.45 |
|  | 1564.65 | 1559.84 | 1573.75 | 1569.80 |
|  | 1562.56 | 1556.66 | 1573.23 | 1569.23 |
|  | 1339.89 | 1337.00 | 1350.57 | 1344.26 |
|  | 1338.82 | 1334.22 | 1350.52 | 1344.02 |
|  | 1335.58 | 1333.51 | 1349.90 | 1343.56 |
| ZPVE | 28.13 | 28.12 | 28.11 | 28.11 |

**Table 4S** Harmonic vibrational frequencies (in cm^−1^) and ZPE (in kcal.mol^−1^) for the transition state, CH_5_.

| mode | M06-2X | ωB97X-D | CCSD(T)^a^ | CCSD(T)^b^ |
| --- | --- | --- | --- | --- |
| ν_1_ | 3237.27 | 3261.96 | 3226.21 | 3218.82 |
| ν_2_ | 3228.26 | 3256.58 | 3221.30 | 3213.94 |
| ν_3_ | 3086.20 | 3106.30 | 3076.51 | 3067.38 |
| ν_4_ | 1735.54 | 1772.88 | 1782.12 | 1778.34 |
| ν_5_ | 1434.53 | 1457.70 | 1445.26 | 1441.89 |
| ν_6_ | 1432.94 | 1449.91 | 1444.73 | 1441.19 |
| ν_7_ | 1120.57 | 1176.67 | 1125.59 | 1119.29 |
| ν_8_ | 1090.70 | 1175.11 | 1124.32 | 1118.59 |
| ν_9_ | 1070.69 | 1114.51 | 1080.45 | 1078.40 |
| ν_10_ | 537.64 | 546.66 | 534.15 | 539.62 |
| ν_11_ | 499.06 | 541.24 | 532.74 | 538.82 |
| ν_i_ | 1538.53i | 1310.27i | 1462.63i | 1469.33 |
| ZPE | 26.41 | 26.96 | 26.58 | 26.52 |

aug-cc-pVTZ^a^ and aug-cc-pVQZ^b^ basis sets

**Table 5S** M062-2X/aug-cc-pVTZ vibrational frequencies (in cm^−1^) and ZPE (in kcal.mol^−1^) for some isotopomers HD, CD_3_, CHD_2_, and CD_4_.

| mode | HD | CD_3_ | CD_4_ | HCD_4_ | DCH_4_ |
| --- | --- | --- | --- | --- | --- |
| ν_1_ | 3866.01 | 2464.76 | 2337.90 | 2406.65 | 3239.21 |
| ν_2_ |  | 2462.78 | 2337.87 | 2399.94 | 3237.91 |
| ν_3_ |  | 2212.83 | 2336.46 | 2195.7 | 3090.91 |
| ν_4_ |  | 1037.21 | 2151.69 | 1717.45 | 1436.04 |
| ν_5_ |  | 1036.06 | 1104.34 | 1046.65 | 1428.98 |
| ν_6_ |  | 324.71 | 1102.31 | 1045.16 | 1399.18 |
| ν_7_ |  |  | 1008.89 | 837.15 | 1120.87 |
| ν_8_ |  |  | 1007.37 | 807.98 | 1118.90 |
| ν_9_ |  |  | 1005.94 | 803.58 | 1020.67 |
| ν_10_ |  |  |  | 443.30 | 498.82 |
| ν_11_ |  |  |  | 407.40 | 483.31 |
| νi |  |  |  | 1145.80i | 1281.13 |
| ZPE | 5.52 | 13.63 | 20.57 | 20.17 | 25.83 |

**Table 6S** ωB97X-D/aug-cc-pVTZ vibrational frequencies (in cm^−1^) and ZPE (in kcal.mol^−1^) for some isotopomers HD, CD_3_, CHD_2_, and CD_4_.

| mode | HD | CD_3_ | CD_4_ | HCD_4_ | DCH_4_ |
| --- | --- | --- | --- | --- | --- |
| ν_1_ | 3840.55 | 2465.76 | 2345.11 | 2407.42 | 3239.21 |
| ν_2_ |  | 2463.23 | 2343.58 | 2404.10 | 3237.91 |
| ν_3_ |  | 2211.31 | 2342.18 | 2197.99 | 3090.91 |
| ν_4_ |  | 1041.65 | 2155.67 | 1775.08 | 1436.04 |
| ν_5_ |  | 1032.02 | 1108.24 | 1049.93 | 1428.98 |
| ν_6_ |  | 382.96 | 1101.24 | 1043.87 | 1399.18 |
| ν_7_ |  |  | 1017.49 | 837.27 | 1120.87 |
| ν_8_ |  |  | 1014.92 | 836.80 | 1118.90 |
| ν_9_ |  |  | 1005.71 | 814.80 | 1020.67 |
| ν_10_ |  |  |  | 434.48 | 498.82 |
| ν_11_ |  |  |  | 431.73 | 483.31 |
| νi |  |  |  | 959.28i | 1281.14i |
| ZPE | 5.49 | 13.71 | 20.63 | 20.34 | 25.83 |

**Table 7S** CCSD(T)/aug-cc-pVTZ vibrational frequencies (in cm^−1^) and ZPE (in kcal.mol^−1^) for the isotopomers HD, CD_3_, CHD_2_, and CD_4_

| HD | CD_3_ | CD_4_ | HCD_4_ | DCH_4_ |
| --- | --- | --- | --- | --- |
| 3812.44 | 2455.85 | 2329.00 | 2394.70 | 3221.52 |
|  | 2455.65 | 2328.79 | 2394.69 | 3221.45 |
|  | 2203.73 | 2328.71 | 2185.90 | 3072.85 |
|  | 1044.16 | 2141.60 | 1762.19 | 1445.30 |
|  | 1043.66 | 1113.30 | 1054.94 | 1444.38 |
|  | 384.71 | 1112.82 | 1054.30 | 1391.49 |
|  |  | 1021.30 | 837.96 | 1104.61 |
|  |  | 1021.22 | 837.79 | 1104.61 |
|  |  | 1020.72 | 809.72 | 1009.79 |
|  |  |  | 436.89 | 480.89 |
|  |  |  | 436.65 | 480.70 |
|  |  |  | 1086.22i | 1451.36i |

**Table 8S** Dual-level rate constants (in cm^3^molecule^−1^s^−1^)^1^ of the forward CH_4_ + H → H_2_ + CH_3_ (*k*_1_) reaction, chemical dynamical calculations, and experimental data.

|  | RPMD^7^ | MCTDH^19^ |  |  | ωB97X-D |  |  |  | M06-2X |  |  |  |  |
| --- | --- | --- | --- | --- | --- | --- | --- | --- | --- | --- | --- | --- | --- |
| T(K) |  |  |  | CVT | CVT/SCT | CVT/μOMT |  | CVT | CVT/SCT | CVT/μOMT |  | [Fit-2001]^22^ | [Fit-2021]^24^ |
| 200 |  |  |  | 2.37E-25 | 1.86E-21 | 3.22E-21 |  | 1.85E-25 | 1.99E-22 | 1.99E-22 |  |  |  |
| 250 |  |  |  | 1.88E-22 | 6.92E-20 | 1.00E-19 |  | 1.65E-22 | 1.17E-20 | 1.17E-20 |  |  |  |
| 300 | 4.73E-19 | 1.9E-19 |  | 1.65E-20 | 1.06E-18 | 1.33E-18 |  | 1.49E-20 | 2.54E-19 | 2.54E-19 |  | 1.90E-19 | 3.10E-19 |
| 350 |  | 2.5E-18 |  | 4.14E-19 | 8.94E-18 | 1.03E-17 |  | 3.81E-19 | 2.85E-18 | 2.85E-18 |  | 2.26E-18 |  |
| 400 | 3.93E-17 | 1.8E-17 |  | 4.73E-18 | 5.00E-17 | 5.45E-17 |  | 4.4E-18 | 1.98E-17 | 1.98E-17 |  | 1.82E-17 | 2.86E-17 |
| 500 | 6.51E-16 | 3.6E-16 |  | 1.52E-16 | 6.84E-16 | 7.10E-16 |  | 1.46E-16 | 3.64E-16 | 3.64E-16 |  | 3.33E-16 | 4.87E-16 |
| 600 | 4.60E-15 | 2.9E-15 |  | 1.63E-15 | 4.60E-15 | 4.69E-15 |  | 1.58E-15 | 2.92E-15 | 2.92E-15 |  | 2.57E-15 | 3.50E-15 |
| 700 | 2.03E-14 |  |  | 9.26E-15 | 1.98E-14 | 2.00E-14 |  | 9.09E-15 | 1.40E-14 | 1.40E-14 |  |  | 1.52E-14 |
| 800 | 6.28E-14 |  |  | 3.54E-14 | 6.30E-14 | 6.34E-14 |  | 3.49E-14 | 4.81E-14 | 4.82E-14 |  | 3.99E-14 | 4.75E-14 |
| 900 | 1.58E-13 |  |  | 1.03E-13 | 1.62E-13 | 1.63E-13 |  | 1.02E-13 | 1.31E-13 | 1.31E-13 |  |  | 1.19E-13 |
| 1000 | 3.31E-13 |  |  | 2.49E-13 | 3.58E-13 | 3.59E-13 |  | 2.47E-13 | 3.00E-13 | 3.00E-13 |  | 2.43E-13 | 2.57E-13 |
| 1500 | 3.72E-12 |  |  | 4.14E-12 | 4.84E-12 | 4.85E-12 |  | 4.14E-12 | 4.44E-12 | 4.44E-12 |  | 3.80E-12 | 3.15E-12 |
| 1550 |  |  |  | 5.03E-12 | 5.82E-12 | 5.83E-12 |  | 5.03E-12 | 5.36E-12 | 5.36E-12 |  |  |  |
| 1800 |  |  |  | 1.16E-11 | 1.29E-11 | 1.29E-11 |  | 1.16E-11 | 1.21E-11 | 1.21E-11 |  | 1.10E-11 | 8.15E-12 |
| 1950 |  |  |  | 1.75E-11 | 1.91E-11 | 1.91E-11 |  | 1.75E-11 | 1.81E-11 | 1.81E-11 |  | 1.71E-11 | 1.20E-11 |
| 2000 | 1.49E-11 |  |  | 1.98E-11 | 5.84E-11 | 2.16E-11 |  | 1.99E-11 | 2.05E-11 | 2.05E-11 |  |  |  |

High-level CCSD(T) harmonic frequencies employed with the VTST-IOC.

**Table 9S** Dual-level rate constants (cm^3^ molecule^−1^s^−1^) of reverse CH_3_ + H_2_ → H + CH_3_ reaction (*k_-_*_1_), previous calculations, and experimental data.

|  |  | ωB97X-D |  |  |  | M06-2X |  | PES^4^ |  | NIST^28^ | NIST^29^ |
| --- | --- | --- | --- | --- | --- | --- | --- | --- | --- | --- | --- |
| T(K) | CVT | CVT/SCT | CVT/μOMT |  | CVT | CVT/SCT | CVT/μOMT | CVT/μOMT |  |  |  |
| 200 | 1.33E-26 | 1.05E-22 | 1.81E-22 |  | 1.06E-26 | 1.14E-23 | 1.14E-23 |  |  |  | 6.78E-26 |
| 250 | 9.89E-24 | 3.63E-21 | 5.27E-21 |  | 8.79E-24 | 6.25E-22 | 6.25E-22 | 4.2E-22 |  |  | 4.79E-23 |
| 300 | 7.90E-22 | 5.06E-20 | 6.36E-20 |  | 7.23E-22 | 1.23E-20 | 1.23E-20 | 1.1E-20 |  | 9.60E-21 | 3.76E-21 |
| 350 | 1.78E-20 | 3.86E-19 | 4.43E-19 |  | 1.67E-20 | 1.25E-19 | 1.25E-19 |  |  |  | 8.42E-20 |
| 400 | 1.84E-19 | 1.95E-18 | 2.12E-18 |  | 1.75E-19 | 7.82E-19 | 7.82E-19 |  |  | 1.10E-18 | 8.62E-19 |
| 500 | 4.90E-18 | 2.21E-17 | 2.29E-17 |  | 4.76E-18 | 1.19E-17 | 1.19E-17 | 1.6E-17 |  | 2.16E-17 |  |
| 600 | 4.4696E-17 | 1.27E-16 | 1.29E-16 |  | 4.41E-17 | 8.15E-17 | 8.15E-17 |  |  | 1.73E-16 |  |
| 700 | 2.24E-16 | 4.78E-16 | 4.83E-16 |  | 2.23E-16 | 3.44E-16 | 3.44E-16 |  |  | 8.16E-16 |  |
| 800 | 7.70E-16 | 1.37E-15 | 1.38E-15 |  | 7.72E-16 | 1.06E-15 | 1.06E-15 | 1.7E-15 |  | 2.74E-15 |  |
| 900 | 2.07E-15 | 3.26E-15 | 3.28E-15 |  | 2.08E-15 | 2.66E-15 | 2.66E-15 |  |  | 7.31E-15 |  |
| 1000 | 4.69E-15 | 6.75E-15 | 6.77E-15 |  | 4.73E-15 | 5.73E-15 | 5.73E-15 | 1.0E-14 |  | 1.65E-14 |  |
| 1500 | 6.79E-14 | 7.93E-14 | 7.94E-14 |  | 6.88E-14 | 7.38E-14 | 7.38E-14 |  |  | 2.44E-13 |  |
| 1550 | 8.21E-14 | 9.49E-14 | 9.50E-14 |  | 8.32E-14 | 8.87E-14 | 8.87E-14 | 1.5E-13 |  |  |  |
| 1800 | 1.88E-13 | 2.09E-13 | 2.09E-13 |  | 1.91E-13 | 1.99E-13 | 1.99E-13 |  |  | 6.80E-13 |  |
| 1950 | 2.85E-13 | 3.12E-13 | 3.12E-13 |  | 2.90E-13 | 2.00E-13 | 3.00E-13 |  |  |  |  |
| 2000 | 3.25E-13 | 3.54E-13 | 3.54E-13 |  | 3.29E-13 | 3.40E-13 | 3.40E-13 | 6.0E-13 |  | 1.18E-12 |  |

High-level CCSD(T) harmonic frequencies employed with the VTST-IOC.

**Table 10S** Dual-level rate constants rate constants (cm^3^ molecule^−1^s^−1^) of the forward H + CD_4_ → HD + CD_3_ (*k*_2_) rate constants and kinetic isotope effect (KIE), *k*_1_/*k*_2._ (High-level CCSD(T) harmonic frequencies).

|  |  | M06-2X |  | KIE |  | ωB97X-D |  |  |  | KIE | PES^14^ | CC/TZ^14^ |
| --- | --- | --- | --- | --- | --- | --- | --- | --- | --- | --- | --- | --- |
| T(K) | CVT | CVT/SCT | CVT/μOMT |  |  | CVT | CVT/SCT |  | CVT/μOMT |  |  |  |
| 250 | 6.63E-27 | 1.68E-24 | 2.46E-22 |  |  | 1.25E-23 | 1.35E-21 |  | 1.35E-21 |  |  |  |
| 300 | 1.52E-21 | 1.13E-20 | 1.13E-20 | 22.48 |  | 1.72E-21 | 3.67E-20 |  | 3.67E-20 | 36.24 | 25.4 | 32.1 |
| 350 | 5.55E-20 | 2.25E-19 | 2.25E-19 | 12.67 |  | 5.98-20 | 5.07E-19 |  | 5.07E-19 | 20.31 |  |  |
| 400 | 8.30E-19 | 2.33E-18 | 2.33E-18 | 8.50 |  | 8.81E-19 | 4.25E-18 |  | 4.25E-18 | 15.82 |  |  |
| 500 | 3.89E-17 | 7.28E-17 | 7.28E-17 | 5.00 |  | 4.05E-17 | 1.05E-16 |  | 1.05E-16 | 6.76 |  |  |
| 600 | 5.35E-16 | 8.13E-16 | 8.13E-16 | 3.59 |  | 5.50E-16 | 1.04E-15 |  | 1.04E-15 | 4.51 | 3.7 | 3.9 |
| 800 | 1.57E-14 | 1.95E-14 | 1.95E-14 | 2.47 |  | 1.60E-14 | 2.25E-14 |  | 2.25E-14 | 2.82 |  |  |
| 1000 | 1.31E-13 | 1.48E-13 | 1.48E-13 | 2.02 |  | 1.32E-13 | 1.63E-13 |  | 1.63E-13 | 2.20 | 2.1 | 2.1 |
| 1500 | 2.63E-12 | 2.75E-12 | 2.75E-12 | 1.61 |  | 2.64E-12 | 2.88E-12 |  | 2.88E-12 | 1.68 | 1.6 | 1.6 |
| 1800 | 7.73E-12 | 7.94E-12 | 7.94E-12 | 1.52 |  | 7.76E-12 | 8.22E-12 |  | 8.22E-12 | 1.57 |  |  |
| 2000 | 1.36E-11 | 1.38E-11 | 1.38E-11 | 1.49 |  | 1.19E-11 | 1.42E-11 |  | 1.42E-11 | 1.52 | 1.5 | 1.5 |

High-level CCSD(T) harmonic frequencies employed with the VTST-IOC.

**Table 11S** Dual-level rate constants (in cm^3^molecule^−1^s^−1^) of D + CH_4_ (R3) → HD + CH_3_ and kinetic isotope effect (k^H+CH4^/k^D+CH4^) previous calculations, and experimental data.

|  |  |  | M06-2X |  |  |  | ωB97X-D |  |  | [4] | [9] | Exp. [30] |
| --- | --- | --- | --- | --- | --- | --- | --- | --- | --- | --- | --- | --- |
| T(K) | CVT | CVT/SCT | CVT/μOMT | KIE |  | CVT | CVT/SCT | CVT/μOMT | KIE |  |  |  |
| 250 | 6.36E-22 | 3.56E-20 | 5.50E-20 | 0.21 |  | 6.79E-22 | 1.81E-19 | 2.74E-19 | 0.36 |  | 0.31 |  |
| 300 | 4.28E-20 | 6.62E-19 | 8.56E-19 | 0.30 |  | 4.49E-20 | 2.28E-18 | 3.02E-18 | 0.44 |  | 0.35 |  |
| 350 | 8.90E-19 | 6.39E-18 | 7.42E-18 | 0.38 |  | 9.23E-19 | 1.73E-17 | 2.06E-17 | 0.50 |  | 0.46 |  |
| 400 | 8.90E-18 | 3.91E-17 | 4.28E-17 | 0.46 |  | 9.14E-18 | 8.84E-17 | 9.88E-17 | 0.55 |  | 0.53 |  |
| 500 | 2.38E-16 | 5.96E-16 | 6.19E-16 | 0.59 |  | 2.42E-16 | 1.05E-15 | 1.10E-15 | 0.65 | 0.6 | 0.60 | 0.8 ± 0.2 |
| 600 | 2.28E-15 | 4.23E-15 | 4.31E-15 | 0.68 |  | 2.29E-15 | 6.40E-15 | 6.57E-15 | 0.71 | 0.7 | 0.64 | 0.9 ± 0.2 |
| 700 | 1.20E-14 | 1.87E-14 | 1.89E-14 | 0.74 |  | 1.21E-14 | 2.57E-14 | 2.60E-14 | 0.77 | 0.8 | 0.69 | 1.0 ± 0.2 |
| 800 | 4.36E-14 | 6.07E-14 | 6.11E-14 | 0.78 |  | 4.36E-14 | 7.77E-14 | 7.84E-14 | 0.81 |  | 0.73 |  |
| 1000 | 2.87E-13 | 3.51E-13 | 3.52E-13 | 0.85 |  | 2.86E-13 | 4.13E-13 | 4.15E-13 | 0.87 |  |  |  |
| 1500 | 4.46E-12 | 4.83E-12 | 4.83E-12 | 0.92 |  | 4.44E-12 | 5.21E-12 | 5.22E-12 | 0.93 |  |  |  |
| 1800 | 1.22E-11 | 1.29E-11 | 1.29E-11 | 0.94 |  | 1.22E-11 | 1.36E-11 | 1.36E-11 | 0.95 |  |  |  |
| 2000 | 2.08E-11 | 2.16E-11 | 2.17E-11 | 0.95 |  | 2.07E-11 | 2.26E-11 | 2.26E-11 | 0.96 |  |  |  |

High-level CCSD(T) harmonic frequencies employed with the VTST-IOC.

**Table 12S** Dual-level rate constants (in cm^3^molecule^−1^s^−1^) of reverse HD + CH_3_ → H + CD_4_ (k_-2_) and kinetic isotope effect (k^H2+CH3^/k^HD+CH3^), previous calculations, and experimental data.

|  |  |  | ωB97X-D |  |  |  |  | M06-2X |  |
| --- | --- | --- | --- | --- | --- | --- | --- | --- | --- |
| T(K) | CVT |  | CVT/SCT | CVT/μOMT | KIE | CVT | CVT/SCT | CVT/μOMT | KIE |
| 200 | 7.11E-27 |  | 1.50E-23 | 1.50E-23 | 12.07 | 6.07E-27 | 1.54E-24 | 1.54E-24 | 7.40 |
| 250 | 4.27E-24 |  | 4.18E-22 | 4.18E-22 | 12.61 | 3.77E-24 | 8.69E-23 | 8.69E-23 | 7.19 |
| 300 | 2.98E-22 |  | 5.90E-21 | 5.90E-21 | 10.79 | 2.72E-22 | 2.02E-21 | 2.02E-21 | 6.09 |
| 350 | 6.21E-21 |  | 4.98E-20 | 4.98E-20 | 8.90 | 5.93E-2 | 2.40E-20 | 2.40E-20 | 5.21 |
| 400 | 6.10E-20 |  | 2.82E-19 | 2.82E-19 | 7.51 | 5.90E-20 | 1.66E-19 | 1.66E-19 | 4.71 |
| 500 | 1.55E-18 |  | 3.92E-18 | 3.92E-18 | 5.84 | 1.52E-18 | 2.85E-18 | 2.85E-18 | 4.18 |
| 600 | 1.40E-17 |  | 2.61E-17 | 2.61E-17 | 4.96 | 1.39E-17 | 2.11E-17 | 2.11E-17 | 3.86 |
| 700 | 7.01E-17 |  | 1.10E-16 | 1.10E-16 | 4.39 | 7.01E-17 | 9.41E-17 | 9.41E-17 | 3.66 |
| 800 | 2.44E-16 |  | 3.41E-16 | 3.41E-16 | 4.04 | 2.45E-16 | 3.05E-16 | 3.05E-16 | 3.46 |
| 1000 | 1.53E-15 |  | 1.88E-15 | 1.88E-15 | 3.60 | 1.54E-15 | 1.75E-15 | 1.75E-15 | 3.27 |
| 1500 | 2.34E-14 |  | 2.55E-14 | 2.55E-14 | 3.11 | 2.37E-14 | 2.48E-14 | 2.48E-14 | 2.98 |
| 1800 | 6.62E-14 |  | 7.01E-14 | 7.01E-14 | 2.98 | 6.71E-14 | 6.89E-14 | 6.89E-14 | 2.88 |
| 2000 | 1.16E-13 |  | 1.21E-13 | 1.21E-13 | 2.58 | 1.17E-13 | 1.20E-13 | 1.20E-13 | 2.83 |

**Table 13S** Dual-level rate constants of reverse CH_3_ + HD → D + CH_4_ reaction (k_-3_) and kinetic isotope effect (k^H2+CH3^/k^HD+CH3^), previous calculations, and experimental data.

|  |  | M062X |  |  |  | wb97X-D |  |  | PES-2008^4^ | MCTDH^4,8^ | Exp.^57^ |
| --- | --- | --- | --- | --- | --- | --- | --- | --- | --- | --- | --- |
| T(K) | CVT | CVT/SCT | CVT/μOMT | KIE | CVT | CVT/SCT | CVT/μOMT | KIE |  |  |  |
| 250 | 3.59E-24 | 2.01E-22 | 3.10E-22 | 2.01 | 3.78E-24 | 1.00E-21 | 1.52E-21 | 3.47 |  |  |  |
| 300 | 2.92E-22 | 4.51E-21 | 5.83E-21 | 2.11 | 3.02E-22 | 1.53E-20 | 2.03E-20 | 3.13 |  |  |  |
| 350 | 6.69E-21 | 4.81E-20 | 5.59E-20 | 2.24 | 6.84E-21 | 1.28E-19 | 1.53E-19 | 2.90 |  |  |  |
| 400 | 7.03E-20 | 3.09E-19 | 3.38E-19 | 2.31 | 7.12E-20 | 6.88E-19 | 7.70E-19 | 2.76 | 2.7 | 2.3 | 2.9 |
| 500 | 1.92E-18 | 4.82E-18 | 5.00E-18 | 2.36 | 1.93E-18 | 8.38E-18 | 8.81E-18 | 2.60 | 2.6 | 2.2 | 2.4 |
| 600 | 1.81E-17 | 3.36E-17 | 3.42E-17 | 2.38 | 1.80E-17 | 5.01E-17 | 5.14E-17 | 2.50 | 2.5 | 2.1 | 2.2 |
| 700 | 9.28E-17 | 1.44E-16 | 1.46E-16 | 2.36 | 9.18E-17 | 1.95E-16 | 1.98E-16 | 2.44 | 2.4 | 2.0 |  |
| 800 | 3.27E-16 | 4.55E-16 | 4.58E-16 | 2.31 | 3.23E-16 | 5.75E-16 | 5.80E-16 | 2.38 |  |  |  |
| 1000 | 2.07E-15 | 2.53E-15 | 2.54E-15 | 2.26 | 2.04E-15 | 2.94E-15 | 2.95E-15 | 2.29 |  |  |  |
| 1500 | 3.18E-14 | 3.45E-14 | 3.45E-14 | 2.14 | 3.1E-14 | 3.67E-14 | 3.68E-14 | 2.16 |  |  |  |
| 1800 | 9.00E-14 | 9.49E-14 | 9.50E-14 | 2.09 | 8.84E-14 | 9.87E-14 | 9.88E-14 | 2.12 |  |  |  |
| 2000 | 1.57E-13 | 1.64E-13 | 1.64E-13 | 2.07 | 1.55E-13 | 1.69E-13 | 1.69E-13 | 2.09 |  |  |  |

^11^(MCTDH) multiconfigurational time-dependent Hartree.

**Table 14S** Dual-level rate constants (in cm^3^molecule^−1^s^−1^) of the forward CH_4_ + H → H_2_ + CH_3_ (*k*_1_) reaction, chemical dynamical calculations, and experimental data.

|  | RPMD^12^ | MCTDH^15^ |  |  | ωB97X-D |  |  |  | M06-2X |  |  |  |  |
| --- | --- | --- | --- | --- | --- | --- | --- | --- | --- | --- | --- | --- | --- |
| T(K) |  |  |  | CVT | CVT/SCT | CVT/μOMT |  | CVT | CVT/SCT | CVT/μOMT |  | Fit-2001^22^ | Fit-2001^24^ |
| 200 |  |  |  | 8.76E-26 | 8.92E-22 | 1.84E-21 |  | 3.48E-25 | 5.99E-22 | 5.99E-22 |  |  |  |
| 250 |  |  |  | 8.48E-23 | 3.40E-20 | 5.51E-20 |  | 2.61E-22 | 2.65E-20 | 2.65E-20 |  |  |  |
| 300 | 4.73E-19 | 1.9E-19 |  | 8.45E-21 | 5.43E-19 | 7.28E-19 |  | 2.20E-20 | 4.87E-19 | 4.87E-19 |  | 1.90E-19 | 3.10E-19 |
| 350 |  | 2.5E-18 |  | 2.31E-19 | 4.84E-18 | 5.75E-18 |  | 5.36E-19 | 4.85E-18 | 4.85E-18 |  | 2.26E-18 |  |
| 400 | 3.93E-17 | 1.8E-17 |  | 2.82E-18 | 2.84E-17 | 3.15E-17 |  | 6.01E-18 | 3.11E-17 | 3.11E-17 |  | 1.82E-17 | 2.86E-17 |
| 500 | 6.51E-16 | 3.6E-16 |  | 9.87E-17 | 4.23E-16 | 4.40E-16 |  | 1.88E-16 | 5.14E-16 | 5.14E-16 |  | 3.33E-16 | 4.87E-16 |
| 600 | 4.60E-15 | 2.9E-15 |  | 1.11E-15 | 3.02E-15 | 3.08E-15 |  | 1.98E-15 | 3.88E-15 | 3.88E-15 |  | 2.57E-15 | 3.50E-15 |
| 700 | 2.03E-14 |  |  | 6.57E-15 | 1.36E-14 | 1.37E-14 |  | 1.11E-14 | 1.79E-14 | 1.79E-14 |  |  | 1.52E-14 |
| 800 | 6.28E-14 |  |  | 2.57E-14 | 4.45E-14 | 4.48E-14 |  | 4.20E-14 | 5.99E-14 | 5.99E-14 |  | 3.99E-14 | 4.75E-14 |
| 900 | 1.58E-13 |  |  | 7.64E-14 | 1.17E-13 | 1.18E-13 |  | 1.22E-13 | 1.60E-13 | 1.60E-13 |  |  | 1.19E-13 |
| 1000 | 3.31E-13 |  |  | 1.86E-13 | 2.63E-13 | 2.64E-13 |  | 2.91E-13 | 3.60E-13 | 3.60E-13 |  | 2.43E-13 | 2.57E-13 |
| 1500 | 3.72E-12 |  |  | 3.21E-12 | 3.71E-12 | 3.72E-12 |  | 4.78E-12 | 5.17E-12 | 5.17E-12 |  | 3.80E-12 | 3.15E-12 |
| 1550 |  |  |  | 3.90E-12 | 4.47E-12 | 4.48E-12 |  | 5.80E-12 | 6.23E-12 | 6.23E-12 |  |  |  |
| 1800 |  |  |  | 9.03E-12 | 9.97E-12 | 9.98E-12 |  | 1.33E-11 | 1.39E-11 | 1.39E-11 |  | 1.10E-11 | 8.15E-12 |
| 1950 |  |  |  | 1.37E-11 | 1.49E-11 | 1.49E-11 |  | 2.00E-11 | 2.08E-11 | 2.08E-11 |  | 1.71E-11 | 1.20E-11 |
| 2000 | 1.49E-11 |  |  | 1.55E-11 | 1.68E-11 | 1.68E-11 |  | 2.270E-11 | 2.36E-11 | 2.36E-11 |  |  |  |

High-level ωB97X-D and M06-2X frequencies used in VTST-IOC.

**Table 15S** Dual-level rate constants rate constants (cm^3^ molecule^−1^s^−1^) of reverse CH_3_ + H_2_ → H + CH_4_ reaction (*k_-_*_1_), chemical dynamical calculations, and experimental data. (High-level ωB97X-D and M06-2X frequencies)

|  |  | ωB97X-D |  |  |  | M06-2X |  | PES^4^ |  | NIST^28^ | NIST^29^ |
| --- | --- | --- | --- | --- | --- | --- | --- | --- | --- | --- | --- |
| T(K) | CVT | CVT/SCT | CVT/μOMT |  | CVT | CVT/SCT | CVT/μOMT | CVT/μOMT |  |  |  |
| 200 | 5.89E-27 | 6.01E-23 | 1.23E-22 |  | 1.06E-26 | 1.14E-23 | 1.14E-23 |  |  |  | 6.78E-26 |
| 250 | 5.15E-24 | 2.07E-21 | 3.35E-21 |  | 8.79E-24 | 6.25E-22 | 6.25E-22 | 4.2E-22 |  |  | 4.79E-23 |
| 300 | 4.58E-22 | 2.9E-20 | 3.95E-20 |  | 7.23E-22 | 1.23E-20 | 1.23E-20 | 1.1E-20 |  | 9.60E-21 | 3.76E-21 |
| 350 | 1.11E-20 | 2.33E-19 | 2.76E-19 |  | 1.67E-20 | 1.25E-19 | 1.25E-19 |  |  |  | 8.42E-20 |
| 400 | 1.21E-19 | 1.22E-18 | 1.35E-18 |  | 1.75E-19 | 7.82E-19 | 7.82E-19 |  |  | 1.10E-18 | 8.62E-19 |
| 500 | 3.46E-18 | 1.48E-17 | 1.54E-17 |  | 4.76E-18 | 1.19E-17 | 1.19E-17 | 1.6E-17 |  | 2.16E-17 |  |
| 600 | 3.30E-17 | 8.94E-17 | 9.11E-17 |  | 4.41E-17 | 8.15E-17 | 8.15E-17 |  |  | 1.73E-16 |  |
| 700 | 1.70E-16 | 3.50E-16 | 3.54E-16 |  | 2.23E-16 | 3.44E-16 | 3.44E-16 |  |  | 8.16E-16 |  |
| 800 | 5.98E-16 | 1.03E-15 | 1.04E-15 |  | 7.72E-16 | 1.06E-15 | 1.06E-15 | 1.7E-15 |  | 2.74E-15 |  |
| 900 | 1.63E-15 | 2.51E-15 | 2.52E-15 |  | 2.08E-15 | 2.66E-15 | 2.66E-15 |  |  | 7.31E-15 |  |
| 1000 | 3.73E-15 | 5.26E-15 | 5.28E-15 |  | 4.73E-15 | 5.73E-15 | 5.73E-15 | 1.0E-14 |  | 1.65E-14 |  |
| 1500 | 5.55E-14 | 6.42E-14 | 6.30E-14 |  | 6.88E-14 | 7.38E-14 | 7.38E-14 |  |  | 2.44E-13 |  |
| 1550 | 6.72E-14 | 7.70E-14 | 7.71E-14 |  | 8.32E-14 | 8.87E-14 | 8.87E-14 | 1.5E-13 |  |  |  |
| 1800 | 1.54E-13 | 1.71E-13 | 1.71E-13 |  | 1.91E-13 | 1.99E-13 | 1.99E-13 |  |  | 6.80E-13 |  |
| 1950 | 2.35E-13 | 2.56E-13 | 2.56E-13 |  | 2.90E-13 | 2.00E-13 | 3.00E-13 |  |  |  |  |
| 2000 | 2.68E-13 | 2.90E-13 | 2.90E-13 |  | 3.29E-13 | 3.40E-13 | 3.40E-13 | 6.0E-13 |  | 1.18E-12 |  |

High-level ωB97X-D and M06-2X frequencies used in VTST-IOC.

**Table 16S** Dual-level rate constants rate constants (cm^3^ molecule^−1^s^−1^) of the forward H + CD_4_ → HD + CD_3_ (k_2_) rate constants and kinetic isotope effects (KIE) (k^H+CH4^/k^H+CD4^)

|  |  | ωB97X-D |  |  |  |  | M06-2X |  |  |  | PES^14^ | CCSD(T)/cc-pVTZ^14^ |
| --- | --- | --- | --- | --- | --- | --- | --- | --- | --- | --- | --- | --- |
| T(K) | CVT | CVT/SCT | CVT/μOMT | KIE |  | CVT | CVT/SCT | CVT/μOMT | KIE |  |  |  |
| 200 | 7.38E-27 | 1.18E-23 | 1.18E-23 | 155.93 |  | 1.11E-26 | 2.03E-24 | 2.03E-24 | 295.07 |  |  |  |
| 250 | 1.16E-23 | 9.04E-22 | 9.04E-22 | 60.95 |  | 1.66E-23 | 3.06E-22 | 3.06E-22 | 86.60 |  |  |  |
| 300 | 1.61E-21 | 2.68E-20 | 2.68E-20 | 27.16 |  | 2.26E-21 | 1.45E-20 | 1.45E-20 | 33.59 |  | 25.4 | 32.1 |
| 350 | 5.68E-20 | 4.00E-19 | 4.00E-19 | 14.38 |  | 7.84E-20 | 2.86E-19 | 2.86E-19 | 16.96 |  |  |  |
| 400 | 8.43E-19 | 3.52E-18 | 3.52E-18 | 8.95 |  | 7.84E-20 | 2.99E-18 | 2.99E-18 | 10.40 |  |  |  |
| 500 | 3.91E-17 | 9.29E-17 | 9.29E-17 | 4.74 |  | 5.47E-17 | 9.71E-17 | 9.71E-17 | 5.29 |  |  |  |
| 600 | 5.35E-16 | 9.55E-16 | 9.55E-16 | 3.22 |  | 7.42E-16 | 1.08E-15 | 1.08E-15 | 3.59 |  | 3.7 | 3.9 |
| 800 | 1.56E-14 | 2.13E-14 | 2.13E-14 | 2.09 |  | 2.15E-14 | 2.59E-14 | 2.59E-14 | 2.31 |  |  |  |
| 1000 | 1.29E-13 | 1.57E-13 | 1.57E-13 | 1.68 |  | 1.77E-13 | 1.96E-13 | 1.96E-13 | 1.84 |  | 2.1 | 2.1 |
| 1500 | 2.60E-12 | 2.81E-12 | 2.81E-12 | 1.32 |  | 3.52E-12 | 3.63E-12 | 3.63E-12 | 1.42 |  | 1.6 | 1.6 |
| 1800 | 7.64E-12 | 8.05E-12 | 8.05E-12 | 2.10 |  | 1.03E-11 | 1.05E-11 | 1.05E-11 | 1.32 |  |  |  |
| 2000 | 1.34E-11 | 1.40E-11 | 1.40E-11 | 1.20 |  | 1.81E-11 | 1.83E-11 | 1.83E-11 | 1.29 |  | 1.5 | 1.5 |

High-level ωB97X-D and M06-2X frequencies used in VTST-IOC.

**Table 17S** Dual-level rate constants (in cm^3^molecule^−1^s^−1^) of D + CH_4_ → HD + CH_3_ (R3) and kinetic isotope effect (k^CH4+H^/k^D+CH4^), previous calculations, and experimental data.

|  |  | ωB97X-D^1^ |  |  |  |  | M06-2X |  |  | [4] | [9] | Exp. [30] |
| --- | --- | --- | --- | --- | --- | --- | --- | --- | --- | --- | --- | --- |
| T(K) | CVT | CVT/SCT | CVT/μOMT | KIE | CVT | CVT/SCT | CVT/μOMT | KIE |  |  |  |  |
| 200 | 1.02E-24 | 7.2498E-21 | 3.6664E-20 | 0.05 | 1.78E-24 | 1.6995E-21 | 2.11E-21 | 0.28 |  |  |  |  |
| 250 | 5.51E-22 | 2.2813E-19 | 6.8595E-19 | 0.08 | 8.76E-22 | 3.0800E-20 | 4.56E-20 | 0.58 |  |  | 0.31 |  |
| 300 | 3.75E-20 | 2.9482E-18 | 5.9930E-18 | 0.12 | 5.62E-20 | 6.1445E-19 | 7.81E-19 | 0.62 |  |  | 0.35 |  |
| 350 | 7.84E-19 | 2.1352E-17 | 3.3603E-17 | 0.17 | 1.13E-18 | 6.3564E-18 | 7.32E-18 | 0.66 |  |  | 0.46 |  |
| 400 | 7.87E-18 | 1.0431E-16 | 1.4006E-16 | 0.23 | 1.10E-17 | 4.0574E-17 | 4.42-17 | 0.70 |  |  | 0.53 |  |
| 500 | 2.11E-16 | 1.1555E-15 | 1.3244E-15 | 0.33 | 2.85E-16 | 6.4271E-16 | 6.68E-16 | 0.77 |  | 0.6 | 0.60 | 0.8 ± 0.2 |
| 600 | 2.03E-15 | 6.7016E-15 | 7.2012E-15 | 0.43 | 2.67E-15 | 4.6310E-15 | 4.73E-15 | 0.82 |  | 0.7 | 0.64 | 0.9 ± 0.2 |
| 700 | 1.07E-14 | 2.5976E-14 | 2.7099E-14 | 0.51 | 1.39E-14 | 2.0640E-14 | 2.09E-14 | 0.86 |  | 0.8 | 0.69 | 1.0 ± 0.2 |
| 800 | 3.89E-14 | 7.6793E-14 | 7.8907E-14 | 0.57 | 5.00E-14 | 6.7067E-14 | 6.76E-14 | 0.89 |  |  | 0.73 |  |
| 1000 | 2.56E-13 | 3.9621E-13 | 4.0160E-13 | 0.66 | 3.24E-13 | 3.8842E-13 | 3.90E-13 | 0.92 |  |  |  |  |
| 1500 | 3.98E-12 | 4.8296E-12 | 4.8506E-12 | 0.77 | 4.97E-12 | 5.3385E-12 | 5.35E-12 | 0.97 |  |  |  |  |
| 1800 | 1.09E-11 | 1.2479E-11 | 1.2513E-11 | 0.79 | 1.36E-11 | 1.4223E-11 | 1.42E-11 | 0.98 |  |  |  |  |
| 2000 | 1.86E-11 | 2.0683E-11 | 2.0728E-11 | 0.81 | 2.30E-11 | 2.3894E-11 | 2.39E-11 | 0.99 |  |  |  |  |

High-level ωB97X-D and M06-2X frequencies used in VTST-IOC.

**Table 18S** Dual-level rate constants (in cm^3^molecule^−1^s^−1^) of reverse HD + CD_3_ → H + CD_4_ (k_-2_),

and kinetic isotope effect (k^H2+CH3^/k^HD+CD3^), previous calculations, and experimental data.

|  |  | ωB97X-D |  |  |  |  | M06-2X |  |
| --- | --- | --- | --- | --- | --- | --- | --- | --- |
| T(K) | CVT | CVT/SCT | CVT/μOMT | KIE | CVT | CVT/SCT | CVT/μOMT | KIE |
| 200 | 7.17E-27 | 1.15E-23 | 1.15E-23 | 10.70 | 1.21E-26 | 2.22E-24 | 2.22E-24 | 5.14 |
| 250 | 4.28E-24 | 3.35E-22 | 3.35E-22 | 10.0 | 6.68E-24 | 1.23E-22 | 1.23E-22 | 5.08 |
| 300 | 2.99E-22 | 4.97E-21 | 4.97E-21 | 7.93 | 4.45E-22 | 2.85E-21 | 2.85E-21 | 4.32 |
| 350 | 6.23E-21 | 4.38E-20 | 4.38E-20 | 6.30 | 8.97E-21 | 3.27E-20 | 3.27E-20 | 3.82 |
| 400 | 6.13E-20 | 2.56E-19 | 2.56E-19 | 5.27 | 8.63E-20 | 2.24E-19 | 2.24E-19 | 3.49 |
| 500 | 1.55E-18 | 3.69E-18 | 3.69E-18 | 4.25 | 2.19E-18 | 3.90E-18 | 3.90E-18 | 3.05 |
| 600 | 1.40E-17 | 2.51E-17 | 2.51E-17 | 3.63 | 1.94E-17 | 2.83E-17 | 2.83E-17 | 2.88 |
| 700 | 7.06E-17 | 1.07E-16 | 1.07E-16 | 3.31 | 9.59E-17 | 1.25E-16 | 1.25E-16 | 2.75 |
| 800 | 2.46E-16 | 3.35E-16 | 3.35E-16 | 3.10 | 3.30E-16 | 3.99E-16 | 3.99E-16 | 2.66 |
| 1000 | 1.54E-15 | 1.86E-15 | 1.86E-15 | 2.83 | 2.04E-15 | 2.26E-15 | 2.26E-15 | 2.53 |
| 1500 | 2.35E-14 | 2.54E-14 | 2.54E-14 | 2.48 | 3.06E-14 | 3.16E-14 | 3.16E-14 | 2.34 |
| 1800 | 6.65E-14 | 7.00E-14 | 7.00E-14 | 2.44 | 8.60E-14 | 8.74E-14 | 8.74E-14 | 2.28 |
| 2000 | 1.16E-13 | 1.21E-13 | 1.21E-13 | 2.40 | 1.50E-13 | 1.51E-13 | 1.51E-13 | 2.25 |

High-level ωB97X-D and M06-2X frequencies used in VTST-IOC.

**Table 19S** Dual-level rate constants of reverse CH_3_ + HD + CH_4_ reaction (k_-3_) and kinetic isotope effect (k^H2+CH3^/k^HD+CH3^), previous calculations, and experimental data (High-level ωB97X-D and M06-2X frequencies).

|  |  | ωb97X-D |  |  |  | M062X |  |  |  | PES-2008^4^ | MCTDH^4,8^ | Exp.^57^ |
| --- | --- | --- | --- | --- | --- | --- | --- | --- | --- | --- | --- | --- |
| T(K) | CVT | CVT/SCT | CVT/μOMT | KIE | CVT | CVT/SCT | CVT/μOMT | KIE |  |  |  |  |
| 200 | 3.3236E-27 | 2.3651E-23 | 1.1961E-22 | 1.03 | 6.2833E-27 | 5.9919E-24 | 7.4484E-24 | 1.53 |  |  |  |  |
| 250 | 2.6119E-24 | 1.0824E-21 | 3.2545E-21 | 1.03 | 4.4352E-24 | 1.5594E-22 | 2.3076E-22 | 2.70 |  |  |  |  |
| 300 | 2.1650E-22 | 1.7041E-20 | 3.4641E-20 | 1.14 | 3.4343E-22 | 3.7578E-21 | 4.7739E-21 | 2.58 |  |  |  |  |
| 350 | 5.0268E-21 | 1.3693E-19 | 2.1549E-19 | 1.28 | 7.6128E-21 | 4.2862E-20 | 4.9343E-20 | 2.53 |  |  |  |  |
| 400 | 5.3120E-20 | 7.0419E-19 | 9.4557E-19 | 1.43 | 7.7815E-20 | 2.8668E-19 | 3.1257E-19 | 2.50 |  | 2.7 | 2.3 | 2.9 |
| 500 | 1.4637E-18 | 7.9989E-18 | 9.1681E-18 | 1.68 | 2.0516E-18 | 4.6244E-18 | 4.8048E-18 | 2.48 |  | 2.6 | 2.2 | 2.4 |
| 600 | 1.3785E-17 | 4.5591E-17 | 4.8989E-17 | 1.86 | 1.8793E-17 | 3.2586E-17 | 3.3253E-17 | 2.45 |  | 2.5 | 2.1 | 2.2 |
| 700 | 7.0850E-17 | 1.7175E-16 | 1.7917E-16 | 1.98 | 9.4787E-17 | 1.4064E-16 | 1.4237E-16 | 2.42 |  | 2.4 | 2.0 |  |
| 800 | 2.5005E-16 | 4.9387E-16 | 5.0747E-16 | 2.06 | 3.3003E-16 | 4.4322E-16 | 4.4681E-16 | 2.37 |  |  |  |  |
| 1000 | 1.5832E-15 | 2.4498E-15 | 2.4831E-15 | 2.13 | 2.0525E-15 | 2.4575E-15 | 2.4680E-15 | 2.32 |  |  |  |  |
| 1500 | 2.4413E-14 | 2.9608E-14 | 2.9737E-14 | 2.12 | 3.0995E-14 | 3.3299E-14 | 3.3350E-14 | 2.22 |  |  |  |  |
| 1800 | 6.9059E-14 | 7.8900E-14 | 7.9117E-14 | 2.16 | 8.7158E-14 | 9.1337E-14 | 9.1428E-14 | 2.17 |  |  |  |  |
| 2000 | 1.2076E-13 | 1.3446E-13 | 1.3475E-13 | 2.15 | 1.5199E-13 | 1.5764E-13 | 1.5776E-13 | 2.15 |  |  |  |  |

High-level ωB97X-D and M06-2X frequencies used in VTST-IOC.
